# Arterial spin labeling MRI to measure peak-exercise calf muscle perfusion reproducibly discriminates peripheral arterial disease from normal

**DOI:** 10.1186/1532-429X-13-S1-P347

**Published:** 2011-02-02

**Authors:** Amy M West, Craig H Meyer, Frederick H Epstein, Jennifer R Hunter, Joseph M DiMaria, John M Christopher, Christopher M Kramer

**Affiliations:** 1 University of Virginia, Charlottesville, VA, USA

## Objective

We hypothesized that arterial spin labeling (ASL) using MRI at 3 Tesla would be a reliable technique for measuring peak exercise calf muscle blood flow in both normal volunteers and patients with PAD and will discriminate between these groups.

## Background

Prior work demonstrated the utility of first-pass gadolinium-enhanced calf muscle perfusion MRI in patients with peripheral arterial disease (PAD). However patients with PAD often have advanced renal disease which prohibits the use of gadolinium-based contrast agents due to the concern for nephrogenic systemic fibrosis. ASL for quantification of calf muscle blood flow could provide a non-contrast alternative in advanced renal disease.

## Methods

PAD patients had symptomatic claudication and an ankle brachial index (ABI) 0.4-0.9. Age-matched normal subjects (NL) had no PAD risk factors. All subjects performed supine plantar flexion exercise using a pedal ergometer until exhaustion or limiting symptoms and were immediately imaged with a flexible calf coil in a Siemens 3T Trio. At end-exercise, 15 averaged arterial spin labeled images were acquired using a pulsed ASL pulse sequence with single-shot echo-planar imaging readouts. Spin labeling was performed using the proximal inversion with control for off-resonance effects (PICORE) technique and the Q2TIPS modification to minimize errors resulting from transit delay. Siemens post-processing relative blood flow images were used to measure perfusion with a region of interest in the calf muscle area with the greatest signal intensity.

## Results

**Figure 1:**
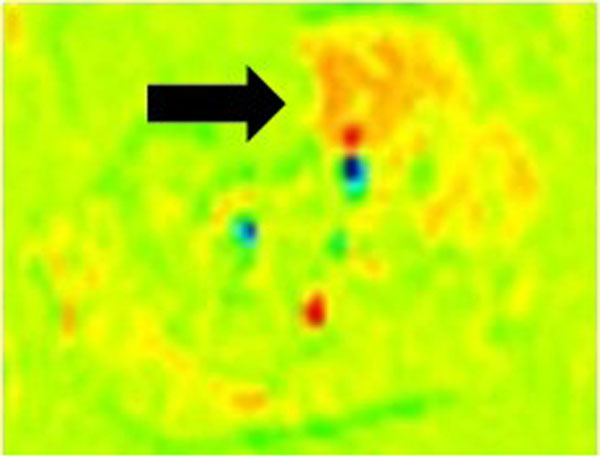
Peak Calf Muscle Perfusion in NL (arrow)

Peak exercise calf perfusion (mean±SD) of 15 NL (age 54±9) was significantly higher than in 15 PAD (age 64±5, ABI 0.70±0.14), see Table 1. Five NL performed exercise matched to PAD (240s) and demonstrated significantly higher perfusion (Table 1). Repeat testing on different days was performed in a group of 12 subjects (5 NL, 7 PAD) with average group perfusion of 65±29mL/min-100g and 66±32mL/min-100g and intra-class correlation coefficient 0.87 (95% CI 0.63-0.96). Figure 1

**Table 1:** Peak Calf Muscle Perfusion in NL and PAD

	NL (n=15)	NL, matched exercise time (n=5)	PAD (n=15)
Exercise time, sec	631±388*	240±0	214±110
Peak perfusion, mL/min-100g	77±23*	84±25*	48±16

* p < 0.01 vs PAD

## Conclusion

ASL is a reproducible non-contrast technique for quantifying peak exercise blood flow in calf muscle. Independent of exercise time, ASL discriminates between NL and PAD. This technique may prove useful for clinical trials of therapies aimed at improving muscle perfusion.

